# Student perceptions toward virtual reality training in dental implant education

**DOI:** 10.7717/peerj.14857

**Published:** 2023-05-05

**Authors:** Yue Huang, Yingwen Hu, Unman Chan, Pengyu Lai, Yueting Sun, Jun Dai, Xin Cheng, Xuesong Yang

**Affiliations:** 1 School of Stomatology, Jinan University, Guangzhou, China; 2 Shanghai VR-Sens Intelligent Technology Co., Ltd, Shanghai, China; 3 Division of Histology and Embryology, Key Laboratory for Regenerative Medicine of the Ministry of Education, Medical College, Jinan University, Guangzhou, China

**Keywords:** Dental education, Dental implant, Usability, Acceptance, Virtual reality (vr)

## Abstract

**Objectives:**

Both the shortage of professional teaching resources and the expensive dental implant supplies impede the effective training of dental undergraduate in implantology. Virtual reality (VR) technology may provide solutions to solve these problems. This pilot study was implemented to explore the usability and acceptance of a VR application in the training of dental implant among dental students at the Jinan University School of Stomatology.

**Methods:**

We designed and developed a VR system with head-mounted displays (HMDs) to assist dental implant training. Undergraduate dental students were invited to experience a 30-minute “Introduction to dental implants” VR-HMDs training module. A total of 119 dental students participated the training. Firstly, the VR interactive training on dental implant was described, illustrated and practiced. Next, a system usability scale (SUS) survey was used to verify the usability and feasibility of the VR application on training dental students. Finally, the participants were given a questionnaire to provide their perceptions and feedback of the usefulness of the VR application for training dental implant skills.

**Results:**

The SUS score was 82.00 ± 10.79, indicating a top 10 percentage ranking of the system’s usabilitys. The participants’ answers to the questionnaire reflected most of them exhibited strong interests in the VR system, with a tendency that the female students were more confident than the male in manipulating the VR system. The participants generally acknowledged the usefulness of VR dental implants, ranking VR value above the traditional laboratory operations, and a preference for using the VR system on learning other skills. They also gave valuable suggestions on VR dental implants for substantial improvement. However, some students were not strongly positive about the VR training in this study, the reason might lie in a more theoretical module was selected for testing, which impacted the students’ ratings.

**Conclusions:**

In this study we revealed the feasibility and usability of VR applications on training dental implant among undergraduate dental students. This pilot study showed that the participants benefited from the dental implant VR training by practicing the skills repeatedly. The feedback from student participants affirmed the advantages and their acceptance of the VR application in dental education. Especially, the VR-based technology is highly conducive to clinical operating skills and surgical procedures-focused training in medical education, indicating that the VR system should be combined with the traditional practice approach in improving dental students’ practical abilities.

## Introduction

The professional training of dentists is quite complex since a qualified dentist must possess knowledge of a variety of clinical competencies (Polychronopoulou & Divaris, 2009), which is acquired through the accumulation of theoretical knowledge, clinical skills, and unique problem-solving abilities (Botelho, Gao & Bhuyan, 2018; Dutã et al., 2011). To meet the requirements of a quickly developing society, dental education has continuously changed in every aspect to prepare competency-based dentists. These changes include areas such as the ability to think critically, apply lifelong learning, scientific research, and knowledge integration, adapt to healthcare needs and a more humanistic environment, and the development of faculty (Change et al., 2006). In addition, patient safety and a focus on quality of care and education are increasingly important, which requires more pre-clinical operational training to acquire the necessary skills.

Major advancements in the sciences, technology, and public health have dramatically impacted dental education (Field, 1995). One such example is the application of medical simulation in dental education (Perry, Bridges & Burrow, 2015). Although the application of simulations in dental education is not new, it has been greatly facilitated when combined with virtual reality (VR) technology, a computer-based simulation of 3-dimensional (3D) images. Consequently, VR technology has been actively incorporated into a variety of dental educational events, teaching, and educational environments in multiple application domains (Correa et al., 2017; Zhang et al., 2020). Especially during the COVID-19 pandemic, dentists are classified as under the very-high-risk category because of the potential of exposure to coronavirus through aerosol-generating procedures, VR works as an effective educational strategy to bridge emergency and ensuring continuity of dental education (Iyer, Aziz & Ojcius, 2020).

VR is a powerful, emerging technology that allows users to experience a high degree of immersion within a 3D environment (Radianti et al., 2020). Because of the significant improvement in affordability and processing power, VR is increasingly used in various educational settings, including remote classroom settings, inorganic and organic chemistry, nanoscience, and biology (Baggio, 2019; Dai et al., 2020; Doutreligne et al., 2014; Lv et al., 2013; Pérez et al., 2015), which has been shown to improve students’ learning outcome *via* an immersive 3D experience. In the scenarios of medical education, VR could supply the optimal conditions to transform a traditional simulation model to a more realistic clinical setting (Levine et al., 2013; Perry, Bridges & Burrow, 2015; Scott et al., 2000; Wilson et al., 1997). The various combinations of VR technologies and dental education have also been employed in preclinical teaching and training for dental students (Buchanan, 2001). For instance, VR techniques combined with haptic device were verified to be satisfactory for the training of needle insertion during dental anesthesia training, including the correct insertion point and depth, as well as the perception of tissues resistances during the insertion (Correa et al., 2017). Virtual simulation education with a jaw simulation model could improve students’ implantology achievements and training (Zhang et al., 2020).

However, there is still some doubtees on the VR applications. For example, in a study which evaluated the students’ acceptance of four different kinds of digital learning technologies (classroom response system, classroom chat, e-lectures and mobile VR), the students evaluated the first three tools favorably before and after usage, except for mobile VR, which saw a substantial drop in perceived usefulness and behavioral intention after 3 months of use (Sprenger & Schwaninger, 2021). They attributed that VR usage in their course did not have a clear link to the exam. Another study about teaching basic surgical tasks with VR headsets showed VR added value to teaching, but only together with high quality traditional teaching methods, the usefulness and usability of VR was experienced more positively (Ojala et al., 2022). It is important to determine the differences between VR and traditional teaching methods and incorporate these methods perfectly in the future. Still more protocols, ideas and users’ experiences and comments should be considered to improve the new techniques widely.

Dental implantation refers to the installation of an endosseous implant *via* a surgical fixture, in which a metal post replaces the root portion of a missing tooth in the jawbone. Although implant dentistry is still a relatively new scientific discipline, it has dramatically expanded and is now practiced all over the world (Dragan et al., 2019). Initially, dental implant education was rooted in clinical training in the form of continuing education courses provided by dentists experienced in implantology (Payant, Williams & Zwemer, 1994). Gradually, this technique has been integrated into the undergraduate dental curriculum system for implant dentistry. At Jinan University College of Stomatology, implant dentistry was separated from prosthetic dentistry and was provided as an optional course in 2017. The course consisted of 36 total teaching credit hours, including 24 credit hours for the theoretical section, and 12 for the practical portion, provided for the fourth-year dental students. The most recently revised education plan of dental students, which will be executed in 2023, has listed implant dentistry as a compulsory course. However, there is still intense debate about whether this technique should be regularly taught and applied among undergraduate dental students (Schweyen et al., 2020). It is commonly thought that more quality-controlled and practice-directed dental implant training is required for improving the education of implant dentistry, especially for novice dentists who are going to work in implant dentistry (Dragan et al., 2019).

VR technology may provide novice dental students and practitioners with effective and repetitive training on dental implants. This training may resolve the practical deficiency that many dentists have when it comes to dental implants, which has been caused by the small size of many dentistry programs or a lack of mentorship. However, the success of technology based learning depends on the attitudes and interactive teaching styles of the faculty, as well as on the experience and attitudes of students with regard to the technology. Therefore, whether the dental students are satisfied with the replacement of VR at their training, and how they perceive the new techniques in learning need more proof to verify. In this study, we developed a novel VR program with immersive head-mounted displays (HMDs) for dental implants, including the crucial steps required for placing a dental implant, as illustrated in Appendix S1. This training was implemented with current undergraduate students at the Jinan University School of Stomatology, Guangzhou, China. Subsequently, we collected feedback regarding the use of the VR application on dental implant training through a follow-up questionnaire survey to the attendees. The aim of this study was to evaluate the usability of VR application in dental implantation and investigate how this technique was perceived and accepted by the dental students in gaining core knowledge and skills in implant dentistry at the phase of preclinical studies.

## Material and Methods

### Participants

**Figure 1:**
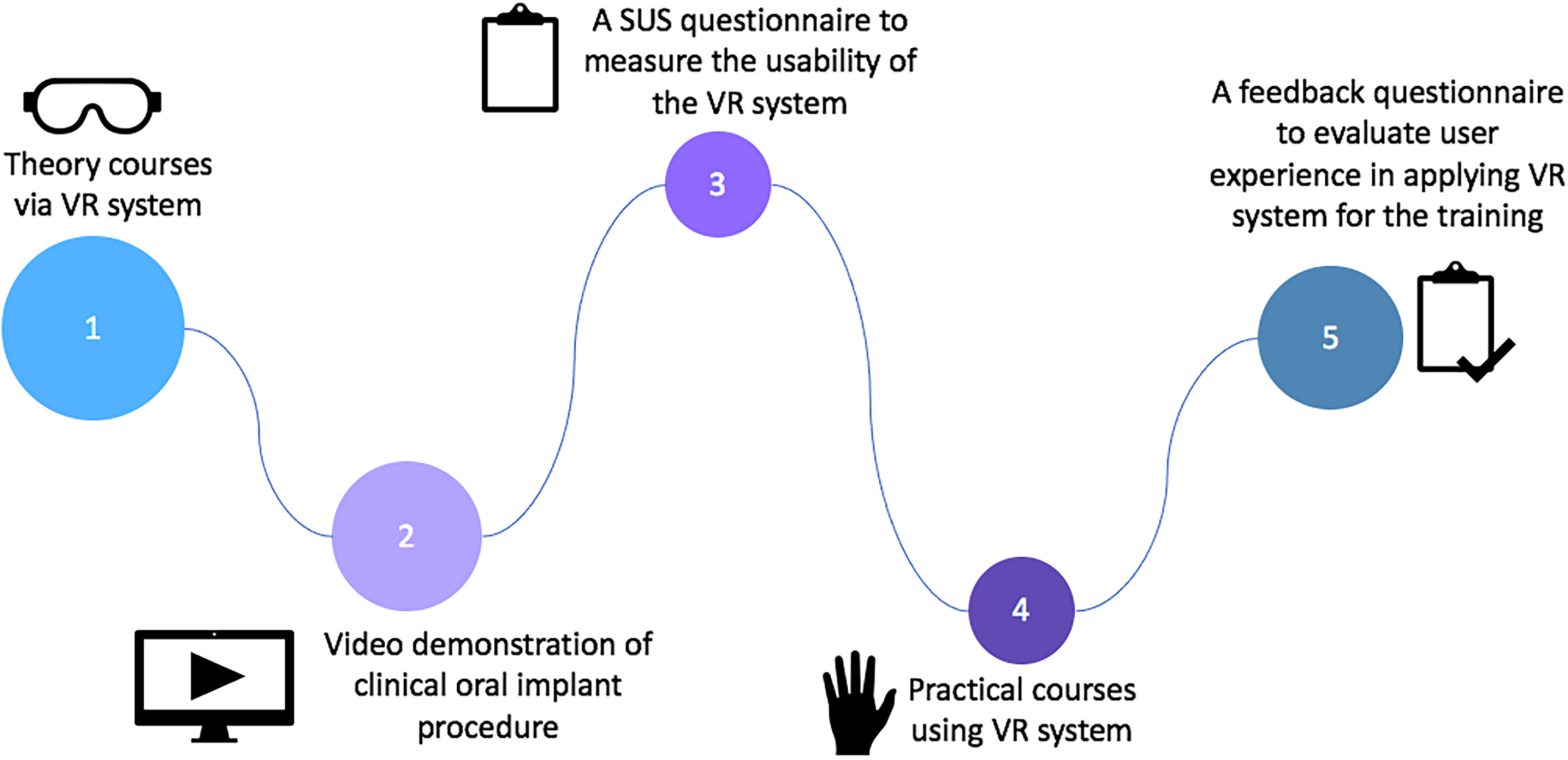
An illustration of the crucial steps in dental implant.

The participants using the dental implant VR technology included undergraduate dental students in their first through fourth years at the Jinan University College of Stomatology, those who had taken dental implant as an optional course or had attended lectures on dental implant previously were excluded from the study. These students were recruited through an on-campus student association. A total of 119 dental students voluntarily signed up for participating this VR training program, no bonus points were awarded for participation. Their average age was 18.99 ± 0.97 years. A survey instrument was designed to collect feedback information about the experiences and usability of the VR technology on the dental implant learning experience of undergraduate dental students. Every participant was asked to answer the questionnaire on SoJump (Ranxing, Changsha, China), an online survey platform in mainland China. Figure 1 is the flow diagram chart of the study.

### VR setup

**Figure 2:**
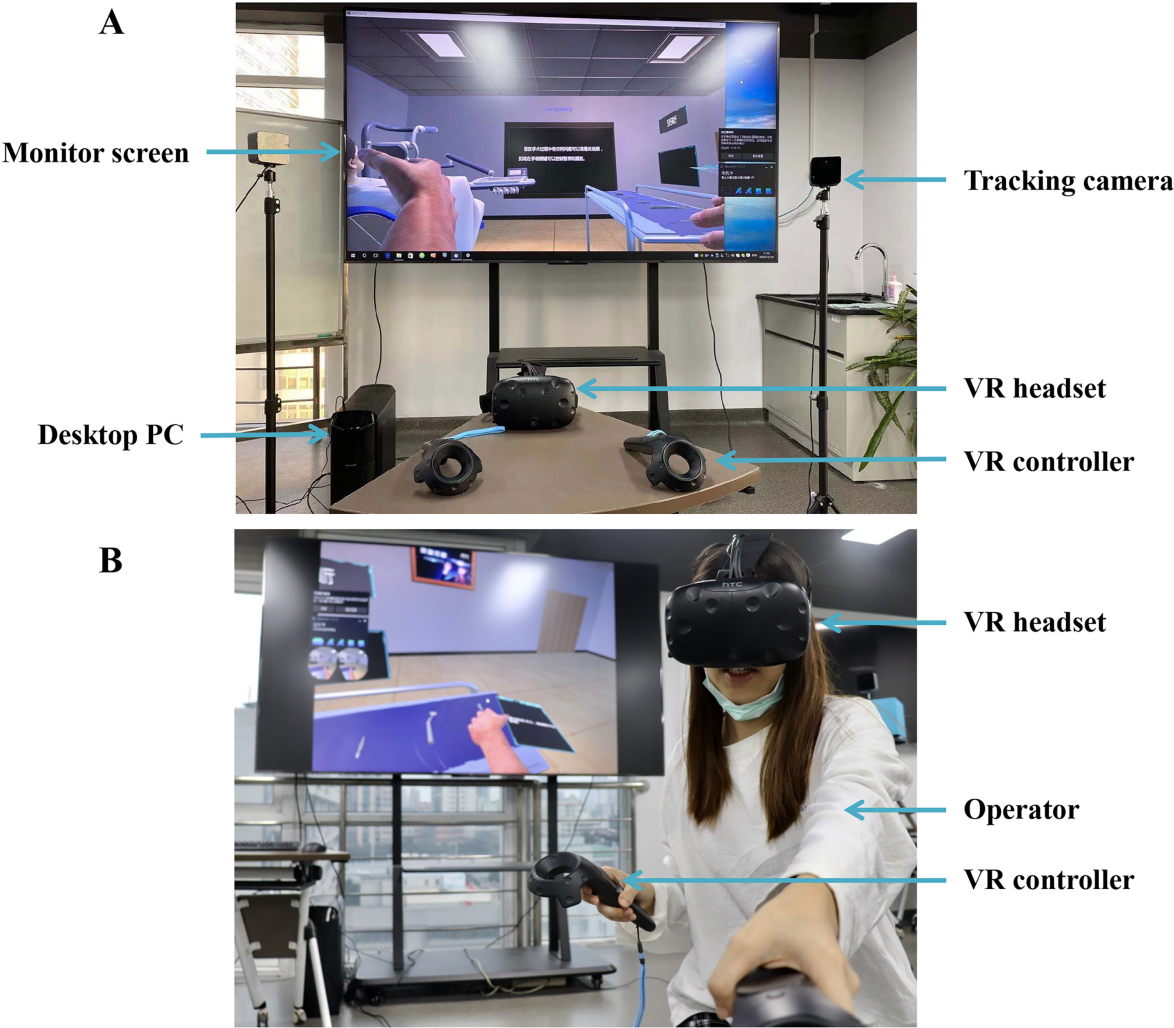
VR setup used for practicing implant dentistry.

The VR system was set up as previously described (Huang et al., 2022). Specifically, the VR system used in this study (Shanghai VR-Sens Intelligent Technology Co. Ltd., Shanghai, China) consisted of a VR interface, a VR headset, two controllers, and two cameras (Fig. 2). The VR system was linked to a conventional gaming computer (CPU: Intel i7-7700 and GPU: NVIDIA GTX1060). The VR interface offered scenarios in virtual reality. Two tracking cameras were used to precisely determine the location of the VR headset. The two controllers were employed to interact with VR objects. As a data visualization tool, custom software (VR-SENS VR Implant Tutorial Software) was utilized. The VR-compatible files were previously generated computationally.

### VR dental implant training and procedures

Students’ dental implant training using VR systems was designed and developed by the first author as a senior dentist and the technical staff of Shanghai VR-Sens Intelligent Technology Co., Ltd., on the basis of a previous report (Payant, Williams & Zwemer, 1994). In this pilot project, we used the “An introduction to dental implant” module as an example to begin the VR application in dentistry. Training sessions lasted approximately half an hour for each participant and were fully supervised by one of the authors to reduce errors. During the operation, the participants were asked to perform the dental implant procedure using a standardized two-handed method. The operating procedure was video recorded. The basic principles of the training included a one-on-one teaching method to provide a VR learning experience. The objectives of the VR session were to make the dental students familiar with the dental implant operating procedure. The VR display of the procedure was shown to the participants using HMDs (Fig. 2).

**Figure 3:**
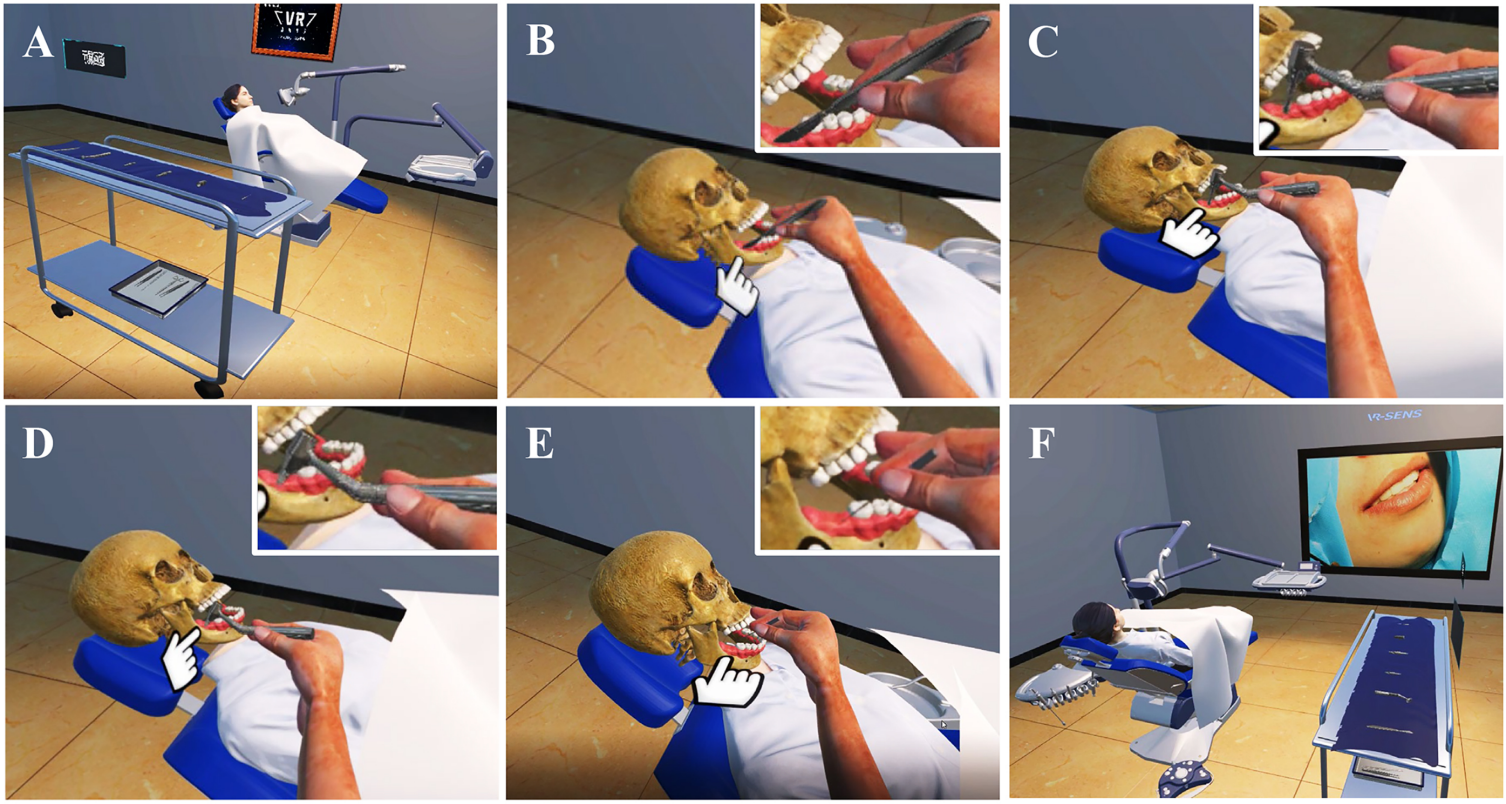
Images of various scenarios in order of dental implant in VR.

Figure 3 showed the main steps of dental implant VR training. Briefly, the student participant wearing the VR-HMD entered the virtual dental implant classroom, according to the developer’s instructions provided, watched a movie to understand the dental implant protocol, and prepared for the implant surgery based on the prompts given (Fig. 3A); the gingival tissue was incised according to the operation tips (Fig. 3B); an implant bore was drilled in the alveolar bone (Fig. 3C); a dental implant was implanted into the hole (Fig. 3D); the wound was stitched after implant surgery (Fig. 3E); and the dental implant training using VR was ended (Fig. 3F).

### Survey questionnaire

In this survey, we utilized two questionnaires. The first survey was based on prior reports of the system usability scale (SUS), in order to determine the users’ acceptability and experience with the VR system and equipment. (Bangor, Kortum & Miller, 2009; Brooke, 1996). The SUS was used to evaluate the VR system, since it has been demonstrated to be a reliable survey method that can be utilized to evaluate the usability of a variety of products and services. SUS is composed of 10 questions in which each response optionally ranges from “strongly disagree” to “strongly agree” (Brooke, 1996). The participant’s scores for each question are summed and then multiplied by 2.5 to transform the original scores of 0 - 40 to a new number between 0 and 100 SUS (Usability.gov). The final SUS score derived from the survey can be considered as an index of the system’s usability and maturity. An SUS score above 68 is considered above average, SUS scores above 80.3 are regarded as within the top tenth percentile.

The second questionnaire collected user feedback on applying VR to dental implants. The questionnaire inquired about the participants’ basic demographics, their VR experience, a system evaluation, and their opinions regarding the use of VR in dental implant teaching. The questionnaire was piloted using 10 college students who did not participate in the main study, to ensure the clarity of the questionnaire. Their feedback led to a panel discussion organized by two senior faculty members to revise and further ascertain the final items to be included in the questionnaire. A total of 18 items were adopted, among which the participants rated 17 items using a 10-point Likert scale, ranging from “0 = strongly disagree” to “9 = strongly agree”. An open-ended question was used to collect suggestions for future development as well as problems or limitations of the VR system as used in clinical dentistry training.

The questionnaire items of the survey instrument were developed in Chinese (see Appendices S2 & S3 for the translated English version) to make it more easily understood by Chinese students. Survey participation was voluntary. All respondents were informed of this study’s aim and general procedures, and were provided with written consent forms about the study and informed that they could withdraw at any time. Confidentiality was assured by keeping the materials de-identified in the transcripts, and the data in this study were only accessible to the authors. This study was approved by the Ethics Committee of Jinan University (No. MJNER202101007).

### Data analysis

The data in this study were processed by SPSS 26.0, including the internal reliability and validity tests, and correlative analysis. The data obtained from the questionnaires were analyzed using Cronbach’s alpha test to determine the internal consistency of the responses. Exploratory factor analyses were employed to determine the factors that reflected the respondents’ attitudes toward VR application in dental implantation. Normality check was done using a Shapiro–Wilk test. Nonparametric analysis (Kruskal-Wallis H tests) was used to test the relationship between participants’ gender and VR experience, and the items of the evaluation of VR system. The results of the descriptive statistics are presented as the mean ± standard deviation (SD) or interquartile range (IQR), and were considered statistically significant when *P* < 0.05.

## Results

### The trainees highly assessed the usability of VR system of dental implantation

The SUS questionnaires were completed by 95 trainees (*i.e.,* the dental students at the Jinan University College of Stomatology) who experienced the VR system, with a response ratio of 79.83%. In this study, the average participant’s score for the survey was 82.00 ± 10.79. Table 1 shows the average score on each item of the SUS questionnaire.

**Table 1:** The evaluation of VR application using system usability scale (SUS, *n* = 95).

**Questions**	**Mean**	**SD**	**Skewness**
1. I think that I would like to use the VR system frequently.	3.93	1.06	0.82
2. I found the VR system unnecessarily complex.	2.26	1.04	−0.60
3. I thought the VR system was easy to use.	3.72	0.93	0.53
4. I think that I would need the support of a technical person to be able to use the VR system.	3.52	0.98	0.43
5. I found the various functions in the VR system were well integrated.	3.61	0.89	0.25
6. I thought there was too much inconsistency in the VR system.	2.44	0.92	−0.51
7. I would imagine that most people would learn to use the VR system very quickly.	3.97	0.96	0.82
8. I found the VR system very cumbersome to use.	2.29	1.00	−0.75
9. I felt very confident using the VR system.	3.56	1.00	0.10
10. I needed to learn a lot of things before I could get going with the VR system.	3.51	1.22	0.34

**Notes.**

Scales, with a 1-5 rating scale from “1=strongly disagree” to “5=strongly agree”.

### The trainees generally acknowledged the usefulness of VR dental implants

To collect the training dental students’ perceptions for dental implant surgery using a VR system, the participants who joined the program were required to answer a feedback questionnaire (Appendix S3). All the participants completed the survey, with a response ratio of 100%. In this study, the Cronbach’s *α* was 0.949, indicating that the reliability and internal consistency of the statistics were sufficient. The participants’ responses are shown in Table 2. Next, three factors were identified by the principal factor analysis about the students’ perceptions of VR application in dental implant surgery module (Table 3): usefulness of VR system (47.93%), prospects of VR application (19.08%) and easiness of VR manipulation (8.25%), which reflected the surveyed respondents’ most differential attitudes toward VR application. Although all three factors could only explain 75.27% of the whole variance, these factors comprise an effective index for evaluating the perceptions of the surveyed students.

**Table 2:** The learner feedback on dental implant training (*n* = 119).

**Questions**	**Mean**	**SD**	**Quartiles**			**Skewness**
			**Q** ^**1**^	**Q** ^**2**^	**Q** ^**3**^	
1. The VR system is easy to use.	4.49	2.40	3	4	5	1.01
2. I am able to study and master VR training programme even without the support of technical person.	6.15	2.23	4	6	8	−0.05
3. I truly perceive the touch and vibration in term of virtual environment during the operation.	4.57	2.32	2	4	6	0.72
4. VR helps me to accurately understand and practise the operation of dental implant surgery clinically.	5.26	2.25	3	5	7	0.31
5. I repeatedly experienced this operation just now.	6.51	2.63	4	7	9	−0.22
6. The 30-minute VR training is enough for me to learn the key points in this module.	5.60	2.39	4	6	8	0.05
7. The VR system is conducive to encourage me to study the content of this module.	4.28	2.34	2	4	5	1.07
8. The application of VR system makes learning process fun.	4.14	2.34	2	3	5	1.21
9. VR system makes the learning more efficiently.	4.39	2.24	3	4	6	0.95
10. In regard to learning outcome, I think that VR system is better than the traditional operation in laboratory.	5.63	2.57	3	5	8	0.26
11. Generally, I think that the VR experience is helpful for my study of dental implant.	4.52	2.16	3	4	5	0.97
12. Regarding to the content of this module, I think that the VR system should be combined with the traditionally practical approach.	3.95	2.37	2	3	5	1.39
13. VR system is very suitable for implementing as an complimentary teaching approach in dental practical sessions.	4.01	2.32	2	3	5	1.29
14. From my experience in utilizing VR system today, I think that the VR system appears to be a mature technology and supplies the real-world environment.	5.18	2.39	3	5	7	0.40
15. I think that the traditional practical teaching in dental education would be replaced by VR system in the future.	6.25	2.43	4	6	8	0.00
16. I would like to spend more time studying dental courses using VR system.	5.32	2.16	4	5	7	0.46
17. After experiencing VR system, I expect that VR system is utilized by other scientific disciplines in teaching and learning.	4.23	2.21	2	4	5	1.07

**Notes.**

Scales, with a 0-9 rating scale from “0 = strongly disagree” to “9 = strongly agree”. Q^1^: quartile at the 25th; Q^2^: quartile at the 50th; Q^3^: quartile at the 75th.

**Table 3:** Summary of the principal factors analysis of the questionnaire on VR application on dental implants (*n* = 119).

Items	Factor 1	Factor 2	Factor 3
12. Regarding to the content of this module, I think that the VR system should be combined with the traditionally practical approach.	0.915		
13. VR system is very suitable for implementing as an complimentary teaching approach in dental practical sessions.	0.914		
8. The application of VR system makes learning process fun.	0.913		
11. Generally, I think that the VR experience is helpful for my study of dental implant.	0.882		
9. VR system makes the learning more efficiently.	0.877		
7. The VR system is conducive to encourage me to study the content of this module.	0.872		
17. After experiencing VR system, I expect that VR system is utilized by other scientific disciplines in teaching and learning.	0.832		
1. The VR system is easy to use.	0.796		
3. I truly perceive the touch and vibration in term of virtual environment during the operation.	0.749		
14. From my experience in utilizing VR system today, I think that the VR system appears to be a mature technology and supplies the real-world environment.	0.609		
6. The 30-minute VR training is enough for me to learn the key points in this module.	0.506		
15. I think that the traditional practical teaching in dental education would be replaced by VR system in the future.		0.853	
10. In regard to learning outcome, I think that VR system is better than the traditional operation in laboratory.		0.683	
16. I would like to spend more time studying dental courses using VR system.		0.669	
5. I repeatedly experienced this operation just now.		0.576	
4. VR helps me to accurately understand and practise the operation of dental implant surgery clinically.		0.564	
2. I am able to study and master VR training programme even without the support of technical person.			0.819
% of Variance	47.93%	19.08%	8.25%
Factors	Usefulness of VR system	Prospects of VR application	Easiness of VR manipulation

**Notes.**

Extraction methods: Principal Component Analysis. Rotation methods: Varimax with Kaiser normalization (KMO = 0.927). Rotation converged in five iterations. The number of factors were determined by the eigenvalues extracted greater than 1. “% of the variance” is the percentage of the variance that the factor can explain of the data set.

### The trainees’ gave valuable suggestions on VR dental implants for improvement

The comments from the open-ended question were qualitatively analyzed. It is possible to categorize the responses into two groups according to the contents: “the suggestions about possible improvements” and “the problems experiencing the VR system”. Among the proposals for potential enhancements include the creation of additional oral surgical procedures using VR technology, the simulation of more realistic oral settings, and the creation of more virtual operations to clearly view the 3D structure of the oral cavity; enhancing immersion by optimizing and enhancing VR scenery, functionality, and features; exhibiting some special complications during dental implants using the VR approach; VR technology could act as a preview of face-to-face sessions; and the application of VR technology should be extended to all medical disciplines. The problems encountered with the VR system included the fact that the technology has not yet reached its full maturity; the fact that the technology was not very user-friendly for people with nearsightedness; the need for technical updates to improve the fluency and comfort of the VR model; the need for more VR-relevant equipment; and the fact that VR could add more value to teaching, although it cannot completely replace high-quality traditional teaching methods.

### The female trainees were more confident in manipulating the VR system

The responses of the participants were then compared based on their gender and VR-experienced/VR-inexperienced status (Table 4). Here, the VR-experienced is defined as the respondents has ever experienced VR no matter for academic usage or for entertainments. The raw data is skewed distributed, therefore nonparametric analysis was used to test the relationship between participants’ gender/VR experience and the items of the evaluation of VR system, since the data has a skewed distribution. The results demonstrated that the items, “I am able to study and master VR training program even without the support of technical person”, and “I repeatedly experienced this operation just now”, exhibited close correlations between the gender differences of participants. Females typically had higher mean ranks (Item 2: 66.65, Item 5: 64.55; *n* = 81) than males (Item 2: 45.83, Item 5: 50.30; *n* = 38). There were no significant differences in the correlations in answering any other questions between male and female participants. Moreover, there were no significant differences between VR-experienced and VR-inexperienced participants according to the correlation analysis.

**Table 4:** The correlation analysis of VR application on dental implant between the learners of different genders and experience (*n* = 119).

**Items**	**Gender**		**Experience**	
	*H*	*P*	*H*	*P*
1. The VR system is easy to use.	0.42	0.517	0.837	0.36
2. I am able to study and master VR training programme even without the support of technical person.	9.581	0.002	1.077	0.299
3. I truly perceive the touch and vibration in term of virtual environment during the operation.	0.151	0.698	0.984	0.321
4. VR helps me to accurately understand and practise the operation of dental implant surgery clinically.	0.026	0.872	0.219	0.64
5. I repeatedly experienced this operation just now.	4.486	0.034	1.192	0.275
6. The 30-minute VR training is enough for me to learn the key points in this module.	2.947	0.086	0.262	0.609
7. The VR system is conducive to encourage me to study the content of this module.	0.001	0.972	0.088	0.766
8. The application of VR system makes learning process fun.	0.125	0.724	0.752	0.386
9. VR system makes the learning more efficiently.	1.32	0.251	0.037	0.847
10. In regard to learning outcome, I think that VR system is better than the traditional operation in laboratory.	0.766	0.381	0.364	0.546
11. Generally, I think that the VR experience is helpful for my study of dental implant.	0.342	0.559	0	0.985
12. Regarding to the content of this module, I think that the VR system should be combined with the traditionally practical approach.	0.017	0.897	0.021	0.884
13. VR system is very suitable for implementing as an complimentary teaching approach in dental practical sessions.	0.375	0.54	0.009	0.926
14. From my experience in utilizing VR system today, I think that the VR system appears to be a mature technology and supplies the real-world environment.	0.038	0.845	0.427	0.514
15. I think that the traditional practical teaching in dental education would be replaced by VR system in the future.	0.576	0.448	0.053	0.819
16. I would like to spend more time studying dental courses using VR system.	2.348	0.125	0.06	0.806
17. After experiencing VR system, I expect that VR system is utilized by other scientific disciplines in teaching and learning.	0.007	0.935	0.225	0.636

**Notes.**

Note: Nonparametric method (Kruskal Wallis H tests) is employed for analysis. *Df* = 1.

## Discussion

In recent decades, the landscape of Chinese dental education system has undergone continuous reforms, in order to cultivate dentists who are qualified to meet the care requirements of the 1.4 billion citizens (Wang, Zhao & Tan, 2017; Wu et al., 2010). However, some problems in the dental education system of China have impeded these efforts; for example, the regional discrepancies in the distribution of dental resources is inconsistent nationwide, the financial and technical support to improve dental students’ practical skills is deficient (Fu et al., 2006). Dental implant is such an example. Regional heterogeneity in implant education is existed not only in China but worldwide.

The application of VR in dental medicine provides solutions to these problems. Using a VR approach, dental students could study theoretical knowledge and simultaneously obtain hands-on experiences that can be used later in clinical work (Joda et al., 2019). By VR-based technology in teaching and learning, dental students or young residents enjoy the advantage of repetitive exercises to master the skills, instead of practicing on patients in a clinic. Assuming no simulation in a virtual environment, our dental students have to observe their instructors’ operations at clinics with very little opportunity to practice themselves. The expectation of having plentiful and superior VR systems to train dental trainees should be met to allow these students to practice until their clinical skills or proficiency satisfy the work requirements at clinics. The application of VR technology may also tremendously abridge teaching costs in dental school and save experienced dentists’ teaching time as well. This type of training allows for the development of a standardized procedure that dental students should be objectively trained in before being allowed to actualize the surgical operation of dental implant on a patient. Therefore, it would be helpful to improve the unequal distribution of educational and academic resources due to the regional economic disparity in China (Wang & Fan, 2004).

VR was first used in healthcare in early 1990s to visualize complex medical structures during surgeries and preoperatively in planning surgeries (Kyaw et al., 2019). The most studied VR application is screen-based display, also known as simulation. In China, the simulation laboratory model is considered common in dental implant education (Zhang et al., 2020; Zhou et al., 2021). However, in this pilot study implemented at the Jinan University School of Stomatology, VR system with HMDs was focused, which is a less-studied form of VR in medical teaching. Compared with regular simulators, the VR system with HMDs is characterized by reliably imitating the sensation of touch so that the operator may experience the objects without having actual physical contact with them, which increasing the feeling of realistic haptics. Except for focusing on the simulated training on psychomotor skills, a clinical scenario has been designed in our dental implantation VR module, to enhance the 3D visualization and to supply the students a more engaging and immersing learning environment, that may foster the teaching and learning of complex medical contents. The VR system is also conducive to developing other basic abilities (*e.g.*, distinguishing anatomical structure, decision-making, accurate diagnosis, etc.), which are also necessary for a qualified dentist in addition to their practical clinical skills.

Although the VR-based approach has been used in the field of endodontics, dental surgery and dental prostheses in some countries (Cayo, Cervantes & Agramonte, 2020; Miki et al., 2016), it has not yet been widely adopted in dental education in mainland China, as it can be expensive and difficult to incorporate into the dental school curriculum (Yeweng, Huang & Ren, 2002; Zhou et al., 2013). Our VR application in dental implants can provide some reference for later design and improvement of such program. The VR system with HMDs was first evaluated using the SUS questionnaire before carrying on the formal survey among the participants on training dental implant. This is a recognized evaluative approach for the usability of a product or service (Bangor, Kortum & Miller, 2009). Our results verified that the VR system was generally well-perceived by the participants in terms of usability, with an average SUS score of 82.00 ± 10.79, suggesting that the score was high enough to accurately rank the VR apparatus among the top tenth percentage based on the above mentioned rules (Brooke, 1996). This SUS score is quite close to another VR-HMD facility developed and reported by Portugal dental educators, IMMPLANT (IMMersive Implant PLANning using a Mobile Touchscreen), with a usability score of 83.91, which revealing the good usability and high learnability of IMMPLANT (Zorzal et al., 2021). Our participants provided positive feedback for their VR experiences, which implies that the traditional teaching methods in a dental laboratory may be replaced by a VR system in the future. Most of the participants acknowledged that “I am able to study and master VR training program even without the support of technical person”, and “I repeatedly experienced this operation just now”. The playfulness of VR exercises is a natural learning approach for this new generation, many of whom have played various games ever since childhood. Nevertheless, this study showed that past VR experience did not affect the participants’ evaluation of the VR application on dental implantation. Meanwhile, female participants showed stronger interest in this new facility than the male without any barrier during usage.

It was worth noting that the results of the questionnaire which collected user feedback on the VR application to dental implants, showed that the students were not overly enthusiastic about this VR-based dental implants training. Some students seemed not to strongly agree that VR could be more efficient and provide sufficient learning. Their acceptance toward the VR training on dental implantation was not so high as the attitudes toward another training module we did almost the same period, the VR training module of orthodontic bracket bonding (Huang et al., 2022). We analyzed the reason might be the different natures of these two modules. The VR-based dental implant training we selected in this study is a general introduction to this technique, but not focusing on any specific implant operational step such as implant placement, so it is more theoretical than the orthodontic bracket bonding module, which is focusing on the training of intraoperative performance. That is, VR is highly conducive to clinical operating skills and surgical procedures-focused training. This phenomenon reminds us that the operational training programs are more suitable to be set up using VR-based techniques, and should be considered first with a limited budget, after all, the VR facilities require specialized equipment and are costly. The drawbacks in the VR application, as identified by the study participants, were valuable contributions that can be used to evaluate the hardware and software of future VR systems. Providing educational services, including excellent facilities to meet students’ learning needs, has always been a challenge for dental schools worldwide. The single application of VR in a dental education would never be sufficient, as the ideal software and corresponding mentoring in each dental specialty requires further improvement.

### Limitations

There were several limitations to our study of VR application for training dental implants. Firstly, we did not elaborate on the acquisition of practical skills between VR participants and nonparticipants who received traditional training, so there was only learning outcome of Kirkpatrick level 1, reactions and perceptions, was reported. VR is currently a new visualization technique, no control was set in this study, and no objective assessment of learning outcomes of VR training has been performed, so there was no evaluation on the quantitative effectiveness of VR-based technology. Secondly, due to the different educational needs, the feedback from the respondents at the Jinan University School of Stomatology may not accurately reflect the educational situation in other dental schools. Thus, further studies are required, to offer an overall conclusion of the efficacy of VR systems application in dental education. The maintenance of the VR-based facilities has not been evaluated. Further research should evaluate the effectiveness of VR in a variety of settings and evaluate outcomes such as cost-effectiveness.

## Conclusion

In this study we revealed the feasibility and usability of VR applications on training dental implant among undergraduate dental students. This pilot study showed that the participants benefited from the dental implant VR training. With the help of VR controllers, dental students were able to interact with a virtual patient and perform a dental implant procedure in the VR system. This program mimicked the real scenarios encountered in clinics. Furthermore, the feedback from student participants affirmed the usability and advantages of the VR application in dental education.

This research has following practical significance for future VR-based dental implant teaching:

1. The repeated preoperative rehearsal and self-assess the work by interactive feedback on a VR system could help students identify the quality of their work and determine skills to improve.

2. Although the VR facilities are costly for hardware and software, HMD can often be easily realized as a headset or glasses, seem to be advantageous to limited budget based on their low-price and mobile nature.

3. When developing VR based education, clinical operational procedures-focused training program should be considered first.

##  Supplemental Information

10.7717/peerj.14857/supp-1 Data S1 Raw data

10.7717/peerj.14857/supp-2 Appendix S1 An illustration of the crucial steps in dental implant (A) Pre-implant inspection, the three-dimensional orientation of the alveolar bone meets the implant placement conditions. (B) Incision of the flap during implantation. (C) Implant placement following cavity preparation. (D) Suture after implant surgery.

10.7717/peerj.14857/supp-3 Appendix S2 The questionnaire to measure user experience in using VR system using SUS

10.7717/peerj.14857/supp-4 Appendix S3 The questionnaire to collect user feedback on applying VR on dental implants

10.7717/peerj.14857/supp-5 Appendis S4 Questionnaire 1 in Chinese

10.7717/peerj.14857/supp-6 Supplemental Information 6 Questionnaire 5 in Chinese

## Additional Information and Declarations

### Data Availability

The following information was supplied regarding data availability: The raw data and statistical processing are available in the Supplementary Files. The Supplemental Video is available in figshare: Chan, Unman (2022): Supplementary Movie1.0.mp4. figshare. Media. https://doi.org/10.6084/m9.figshare.20449641.v1.

## References

[ref-1] Baggio A (2019). Educational technology: a revolution in the didactic milieu. Understanding the originations of the phenomenon through the innovation process of Tel Aviv University. Master’s Degree Thesis.

[ref-2] Bangor A, Kortum P, Miller J (2009). Determining what individual SUS scores mean: Adding an adjective rating scale. Journal of Usability Studies.

[ref-3] Botelho M, Gao X, Bhuyan S (2018). An analysis of clinical transition stresses experienced by dental students: a qualitative methods approach. European Journal of Dental Education.

[ref-4] Brooke J (1996). Sus: a ‘quick and dirty’ usability. Usability evaluation in industry.

[ref-5] Buchanan JA (2001). Use of simulation technology in dental education. Journal of Dental Education.

[ref-6] Cayo C, Cervantes L, Agramonte R (2020). VR systems in dental education. British Dental Journal.

[ref-7] Haden NK, Andrieu SC, Chadwick DG, Chmar JE, Cole JR, George MC, Glickman GN, Glover JF, Goldberg JS, Change ACo, Education IiD (2006). The dental education environment. Journal of dental education.

[ref-8] Correa CG, Machado M, Ranzini E, Tori R, Nunes FLS (2017). Virtual reality simulator for dental anesthesia training in the inferior alveolar nerve block. Journal of Applied Oral Science.

[ref-9] Dai R, Laureanti JA, Kopelevich M, Diaconescu PL (2020). Developing a virtual reality approach toward a better understanding of coordination chemistry and molecular orbitals. Journal of Chemical Education.

[ref-10] Doutreligne S, Cragnolini T, Pasquali S, Derreumaux P, Baaden M (2014). UnityMol: interactive scientific visualization for integrative biology.

[ref-11] Dragan IF, Pirc M, Rizea C, Yao J, Acharya A, Mattheos N (2019). A global perspective on implant education: cluster analysis of the first dental implant experience of dentists from 84 nationalities. European Journal of Dental Education.

[ref-12] Dutã M, Amariei CI, Bogdan CM, Popovici DM, Ionescu N, Nuca CI (2011). An overview of virtual and augmented reality in dental education. Oral Health and Dental Management.

[ref-13] Field MJ (1995). Dental education at the crossroads: challenges and change.

[ref-14] Fu Y, Ling J, Jiang B, Yin H (2006). Perspectives on dental education in mainland China. International Dental Journal.

[ref-15] Huang Y, Cheng X, Chan U, Zheng L, Hu Y, Sun Y, Lai P, Dai J, Yang X (2022). Virtual reality approach for orthodontic education at School of Stomatology, Jinan University. Journal of Dental Education.

[ref-16] Iyer P, Aziz K, Ojcius DM (2020). Impact of COVID-19 on dental education in the United States. Journal of Dental Education.

[ref-17] Joda T, Gallucci G, Wismeijer D, Zitzmann N (2019). Augmented and virtual reality in dental medicine: a systematic review. Computers in Biology and Medicine.

[ref-18] Kyaw BM, Saxena N, Posadzki P, Vseteckova J, Nikolaou CK, George PP, Divakar U, Masiello I, Kononowicz AA, Zary N, Tudor Car L (2019). Virtual reality for health professions education: systematic review and meta-analysis by the digital health education collaboration. Journal of Medical Internet Research.

[ref-19] Levine AI, De Maria Jr S, Schwartz AD, Sim AJ (2013). The comprehensive textbook of healthcare simulation.

[ref-20] Lv Z, Tek A, Da Silva F, Empereur-Mot C, Chavent M, Baaden M (2013). Game on, science-how video game technology may help biologists tackle visualization challenges. PLOS ONE.

[ref-21] Miki T, Iwai T, Kotani K, Dang J, Sawada H, Miyake M (2016). Development of a virtual reality training system for endoscope-assisted submandibular gland removal. Journal of Cranio-Maxillofacial Surgery.

[ref-22] Ojala S, Sirola J, Nykopp T, Kroger H, Nuutinen H (2022). The impact of teacher’s presence on learning basic surgical tasks with virtual reality headset among medical students. Medical Education Online.

[ref-23] Payant L, Williams J, Zwemer J (1994). Survey of dental implant practice. The Journal of Oral Implantology.

[ref-24] Pérez S, Tubiana T, Imberty A, Baaden M (2015). Three-dimensional representations of complex carbohydrates and polysaccharides—sweetunityMol: a video game-based computer graphic software. Glycobiology.

[ref-25] Perry S, Bridges SM, Burrow MF (2015). A review of the use of simulation in dental education. Simulation in Healthcare.

[ref-26] Polychronopoulou A, Divaris K (2009). Dental students’ perceived sources of stress: a multi-country study. Journal of Dental Education.

[ref-27] Radianti J, Majchrzak TA, Fromm J, Wohlgenannt I (2020). A systematic review of immersive virtual reality applications for higher education: Design elements, lessons learned, and research agenda. Computers & Education.

[ref-28] Schweyen R, Al-Nawas B, Arnold C, Hey J (2020). A cross-sectional survey of attitudes towards education in implant dentistry in the undergraduate dental curriculum. International Journal of Implant Dentistry.

[ref-29] Scott DJ, Bergen PC, Rege RV, Laycock R, Tesfay ST, Valentine RJ, Euhus DM, Jeyarajah DR, Thompson WM, Jones DB (2000). Laparoscopic training on bench models: better and more cost effective than operating room experience?. Journal of the American College of Surgeons.

[ref-30] Sprenger DA, Schwaninger A (2021). Technology acceptance of four digital learning technologies (classroom response system, classroom chat, e-lectures, and mobile virtual reality) after three months’ usage. International Journal of Educational Technology in Higher Education.

[ref-31] Wang X, Fan G (2004). Analysis on the regional disparity in China and the influential factors. Economic Research Journal.

[ref-32] Wang Y, Zhao Q, Tan Z (2017). Current differences in dental education between Chinese and Western models. European Journal of Dental Education.

[ref-33] Wilson M, Middlebrook A, Sutton C, Stone R, McCloy R (1997). MIST VR: a virtual reality trainer for laparoscopic surgery assesses performance. Annals of the Royal College of Surgeons of England.

[ref-34] Wu Z, Zhang Z, Jiang X, Guo L (2010). Comparison of dental education and professional development between mainland China and North America. European Journal of Dental Education.

[ref-35] Yeweng S, Huang S, Ren L (2002). Orthodontics in China. Journal of Orthodontics.

[ref-36] Zhang B, Li S, Gao S, Hou M, Chen H, He L, Li Y, Guo Y, Wang E, Cao R, Cheng J, Li R, Zhang K (2020). Virtual versus jaw simulation in Oral implant education: a randomized controlled trial. BMC Medical Education.

[ref-37] Zhou F, Tang C, Wang D, Zhu Y (2013). Discussion on better conjunction between postgraduate and undergraduate education of or-thodontics in China. Chinese Journal of Medical Education Research.

[ref-38] Zhou Y, Chen W, Zhao X, He B, Huang W, Wu D, Chen J (2021). Application evaluation of virtual reality technology in dental implant training: a new dental implant training system: a CONSORT-compliant trial. Medicine.

[ref-39] Zorzal ER, Paulo SF, Rodrigues P, Mendes JJ, Lopes DS (2021). An immersive educational tool for dental implant placement: a study on user acceptance. International Journal of Medical Informatics.

