# Supplementary figures and images for: Student perceptions toward virtual reality training in dental implant education
Source: PeerJ. 2023 May 5;11:e14857. doi: 10.7717/peerj.14857 (PMC10166074; doi:10.7717/peerj.14857)

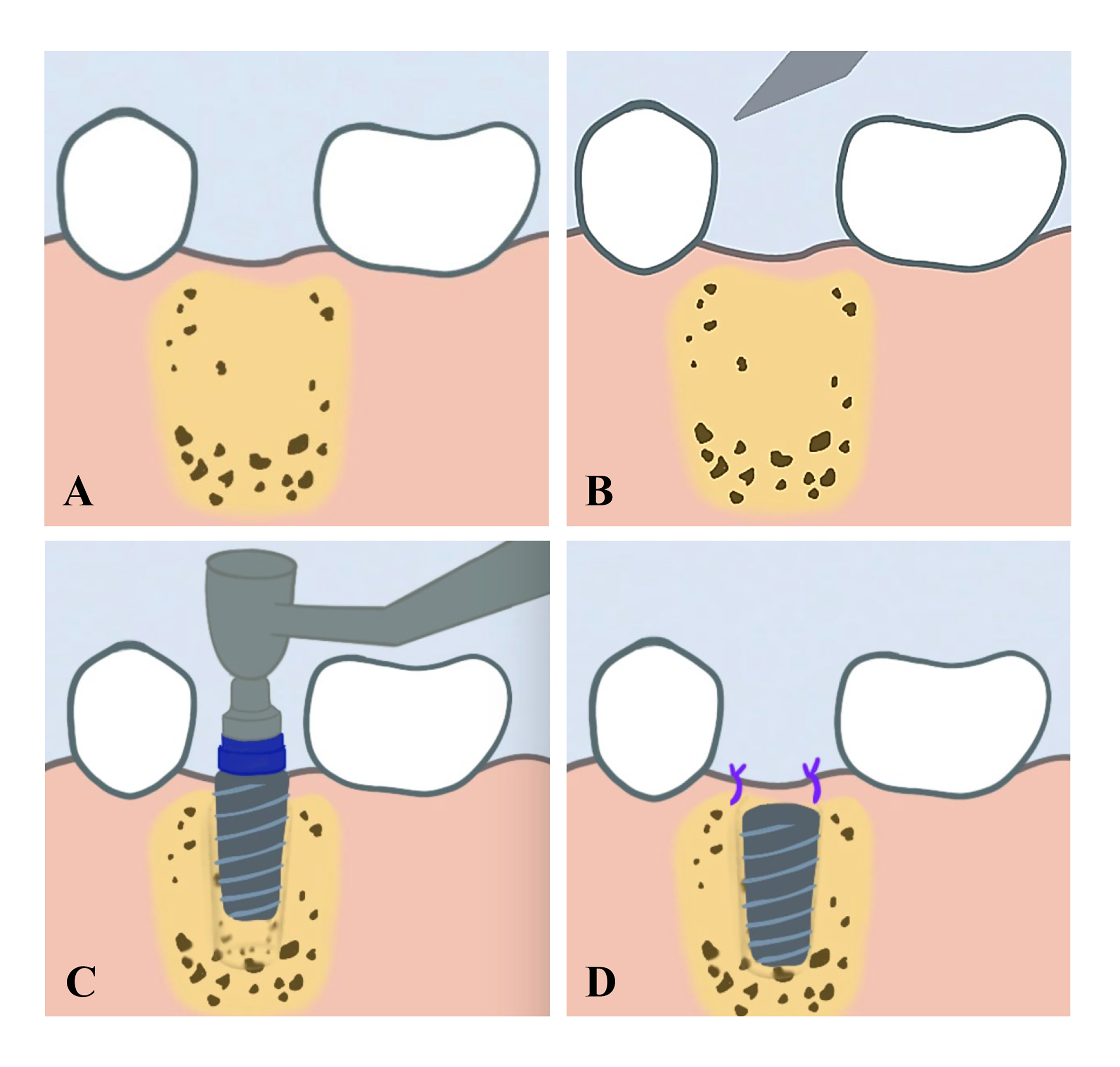

Supplement: Appendix S1 — (A) Pre-implant inspection, the three-dimensional orientation of the alveolar bone meets the implant placement conditions. (B) Incision of the flap during implantation. (C) Implant placement following cavity preparation. (D) Suture after implant surgery. [file peerj-11-14857-s002.tif]
