# Supplementary material for: Student perceptions toward virtual reality training in dental implant education
Source: PeerJ. 2023 May 5;11:e14857. doi: 10.7717/peerj.14857 (PMC10166074; doi:10.7717/peerj.14857)
Supplement: Appendix S2 [file peerj-11-14857-s003.docx]

**A SUS questionnaire to measure the user experience in using VR system**

We sincerely thank you for taking part in this survey on evaluating the participants’ experience in implementing virtual reality (VR) system and equipment. The results will provide useful information to improve the learning outcome of dental education by means of utilizing VR as teaching resource. The survey adopted the “system usability scale (SUS) – it provides a reliable tool for measuring the usability”, which was initially created by John Brooke in 1986 ^[1](#_ENREF_1), [2](#_ENREF_2)^. The questionnaire contains 10 questions with five response options (**from strongly disagree to strongly agree**) for respondents, and takes about 2 minutes to complete. There are no standard answers for these questions. Please answer them based on your own experience of using the VR system and equipment. We pledge to keep all data anonymous, and personal information will not be disclosed. Thank you very much for your participation.

1. I think that I would like to use the VR system frequently. [single choice] *

| ○1 | ○2 | ○3 | ○4 | ○5 |
| --- | --- | --- | --- | --- |

2. I found the VR system unnecessarily complex. [single choice] *

| ○1 | ○2 | ○3 | ○4 | ○5 |
| --- | --- | --- | --- | --- |

3. I thought the VR system was easy to use. [single choice] *

| ○1 | ○2 | ○3 | ○4 | ○5 |
| --- | --- | --- | --- | --- |

4. I think that I would need the support of a technical person to be able to use the VR system. [single choice] *

| ○1 | ○2 | ○3 | ○4 | ○5 |
| --- | --- | --- | --- | --- |

5. I found the various functions in the VR system were well integrated. [single choice] *

| ○1 | ○2 | ○3 | ○4 | ○5 |
| --- | --- | --- | --- | --- |

6. I thought there was too much inconsistency in the VR system. [single choice] *

| ○1 | ○2 | ○3 | ○4 | ○5 |
| --- | --- | --- | --- | --- |

7. I would imagine that most people would learn to use the VR system very quickly. [single choice] *

| ○1 | ○2 | ○3 | ○4 | ○5 |
| --- | --- | --- | --- | --- |

8. I found the VR system very cumbersome to use. [single choice] *

| ○1 | ○2 | ○3 | ○4 | ○5 |
| --- | --- | --- | --- | --- |

9. I felt very confident using the VR system. [single choice] *

| ○1 | ○2 | ○3 | ○4 | ○5 |
| --- | --- | --- | --- | --- |

10. I needed to learn a lot of things before I could get going with the VR system. [single choice] *

| ○1 | ○2 | ○3 | ○4 | ○5 |
| --- | --- | --- | --- | --- |

References

[1] Brooke J: Sus: a “quick and dirty’usability. Usability evaluation in industry 1996, 189.

[2] Bangor A, Kortum P, Miller J: Determining what individual SUS scores mean: Adding an adjective rating scale. Journal of usability studies 2009, 4:114-23.
