# Supplementary material for: Student perceptions toward virtual reality training in dental implant education
Source: PeerJ. 2023 May 5;11:e14857. doi: 10.7717/peerj.14857 (PMC10166074; doi:10.7717/peerj.14857)
Supplement: Appendix S3 [file peerj-11-14857-s004.docx]

**A questionnaire to collect users’ feedback on applying VR on dental implants**

We sincerely thank you for taking part in this pilot dental implant training using virtual reality (VR) system and equipment. This survey aims at the experiences of student participants on implementing VR in dental implant practice. The result will provide the useful information for improving teaching effect of dental implant education by means of utilizing VR as a teaching resource. The questionnaire contains 17 questions with ten response options (from strongly disagree to strongly agree) and 1 open-ended question for respondents, and takes about 5 minutes to complete. There are no standard answers for these questions. Please answer them based on your own experience of using the VR system and equipment. We pledge to keep all data anonymous, and personal information will not be disclosed. Thank you very much again for your participation.

1. Your age? [Please fill in the space below] *

_________________________________

2. Your grade? [Single choice] *

| ○ The first year |
| --- |
| ○ The second year |
| ○ The third year |
| ○ The fourth year |

3. Your gender? [Single choice] *

| ○ Male |
| --- |
| ○ Female |

4. Have you ever used VR equipment before? [Single choice] *

| ○ Yes |
| --- |
| ○ No |

**Note: In regard to the “dental implant” you experienced in VR system, answer the following questions based on the response options (0 = strongly disagree; 9 = strongly agree)**

**Part 1. Practice experience**

1. The VR system is easy to use. [Single choice] *

| ○ 0 | ○ 1 | ○ 2 | ○ 3 | ○ 4 | ○ 5 | ○ 6 | ○ 7 | ○ 8 | ○ 9 |
| --- | --- | --- | --- | --- | --- | --- | --- | --- | --- |

1. I am able to study and master VR training programme even without the support of technical person. [Single choice] *

| ○ 0 | ○ 1 | ○ 2 | ○ 3 | ○ 4 | ○ 5 | ○ 6 | ○ 7 | ○ 8 | ○ 9 |
| --- | --- | --- | --- | --- | --- | --- | --- | --- | --- |

7. I truly perceive the touch and vibration in term of virtual environment during the operation. [Single choice] *

| ○ 0 | ○ 1 | ○ 2 | ○ 3 | ○ 4 | ○ 5 | ○ 6 | ○ 7 | ○ 8 | ○ 9 |
| --- | --- | --- | --- | --- | --- | --- | --- | --- | --- |

8. VR helps me to accurately understand and practise the operation of dental implant surgery clinically. [Single choice] *

| ○ 0 | ○ 1 | ○ 2 | ○ 3 | ○ 4 | ○ 5 | ○ 6 | ○ 7 | ○ 8 | ○ 9 |
| --- | --- | --- | --- | --- | --- | --- | --- | --- | --- |

9. I repeatedly experienced this operation just now. [Single choice] *

| ○ 0 | ○ 1 | ○ 2 | ○ 3 | ○ 4 | ○ 5 | ○ 6 | ○ 7 | ○ 8 | ○ 9 |
| --- | --- | --- | --- | --- | --- | --- | --- | --- | --- |

10. The 30-minute VR training is enough for me to learn the key points in this module. [Single choice] *

| ○ 0 | ○ 1 | ○ 2 | ○ 3 | ○ 4 | ○ 5 | ○ 6 | ○ 7 | ○ 8 | ○ 9 |
| --- | --- | --- | --- | --- | --- | --- | --- | --- | --- |

**Part 2. Systematic assessment**

11. The VR system is conducive to encourage me to study the content of this module. [Single choice] *

| ○ 0 | ○ 1 | ○ 2 | ○ 3 | ○ 4 | ○ 5 | ○ 6 | ○ 7 | ○ 8 | ○ 9 |
| --- | --- | --- | --- | --- | --- | --- | --- | --- | --- |

12. The application of VR system makes learning process fun. [Single choice] *

| ○ 0 | ○ 1 | ○ 2 | ○ 3 | ○ 4 | ○ 5 | ○ 6 | ○ 7 | ○ 8 | ○ 9 |
| --- | --- | --- | --- | --- | --- | --- | --- | --- | --- |

13. VR system makes the learning more efficiently. [Single choice] *

| ○ 0 | ○ 1 | ○ 2 | ○ 3 | ○ 4 | ○ 5 | ○ 6 | ○ 7 | ○ 8 | ○ 9 |
| --- | --- | --- | --- | --- | --- | --- | --- | --- | --- |

14. In regard to learning outcome, I think that VR system is better than the traditional operation in laboratory. [Single choice] *

| ○ 0 | ○ 1 | ○ 2 | ○ 3 | ○ 4 | ○ 5 | ○ 6 | ○ 7 | ○ 8 | ○ 9 |
| --- | --- | --- | --- | --- | --- | --- | --- | --- | --- |

15. Generally, I think that the VR experience is helpful for my study of dental implant. [Single choice] *

| ○ 0 | ○ 1 | ○ 2 | ○ 3 | ○ 4 | ○ 5 | ○ 6 | ○ 7 | ○ 8 | ○ 9 |
| --- | --- | --- | --- | --- | --- | --- | --- | --- | --- |

**Part 3. Your opinion**

16. Regarding to the content of this module, I think that the VR system should be combined with the traditionally practical approach. [Single choice] *

| ○ 0 | ○ 1 | ○ 2 | ○ 3 | ○ 4 | ○ 5 | ○ 6 | ○ 7 | ○ 8 | ○ 9 |
| --- | --- | --- | --- | --- | --- | --- | --- | --- | --- |

17. VR system is very suitable for implementing as an complimentary teaching approach in dental practical sessions. [Single choice] *

| ○ 0 | ○ 1 | ○ 2 | ○ 3 | ○ 4 | ○ 5 | ○ 6 | ○ 7 | ○ 8 | ○ 9 |
| --- | --- | --- | --- | --- | --- | --- | --- | --- | --- |

18. From my experience in utilizing VR system today, I think that the VR system appears to be a mature technology and supplies the real-world environment. [Single choice] *

| ○ 0 | ○ 1 | ○ 2 | ○ 3 | ○ 4 | ○ 5 | ○ 6 | ○ 7 | ○ 8 | ○ 9 |
| --- | --- | --- | --- | --- | --- | --- | --- | --- | --- |

19. I think that the traditional practical teaching in dental education would be replaced by VR system in the future. [Single choice] *

| ○ 0 | ○ 1 | ○ 2 | ○ 3 | ○ 4 | ○ 5 | ○ 6 | ○ 7 | ○ 8 | ○ 9 |
| --- | --- | --- | --- | --- | --- | --- | --- | --- | --- |

20. I would like to spend more time studying dental courses using VR system. [Single choice] *

| ○ 0 | ○ 1 | ○ 2 | ○ 3 | ○ 4 | ○ 5 | ○ 6 | ○ 7 | ○ 8 | ○ 9 |
| --- | --- | --- | --- | --- | --- | --- | --- | --- | --- |

21. After experiencing VR system, I expect that VR system is utilized by other scientific disciplines in teaching and learning. [Single choice] *

| ○ 0 | ○ 1 | ○ 2 | ○ 3 | ○ 4 | ○ 5 | ○ 6 | ○ 7 | ○ 8 | ○ 9 |
| --- | --- | --- | --- | --- | --- | --- | --- | --- | --- |

22. In your opinion, what further applications of VR technology in dental education could be expected in the future? [Please fill in the space below] *

_________________________________
