# Supplementary material for: Student perceptions toward virtual reality training in dental implant education
Source: PeerJ. 2023 May 5;11:e14857. doi: 10.7717/peerj.14857 (PMC10166074; doi:10.7717/peerj.14857)
Supplement: Appendis S4 [file peerj-11-14857-s005.docx]

**关于体验者对VR设备评价的调查问卷**

十分感谢您参与我们VR设备的体验。本次问卷调查将用于完善我们VR设备，提高教学质量。请按照**（1：非常不同意，5：非常同意）**等级，给你刚刚体验过的VR评分。问卷一共10道题，大概需要您2分钟时间完成。再次感谢您的参与。

1. 我认为只要我学习的课程有VR设备，我就会选择使用VR。 [单选题] *

| 非常不同意 | ○1 | ○2 | ○3 | ○4 | ○5 | 非常同意 |
| --- | --- | --- | --- | --- | --- | --- |

2. 我觉得我刚体验过的VR存在复杂且没有必要的功能。 [单选题] *

| 非常不同意 | ○1 | ○2 | ○3 | ○4 | ○5 | 非常同意 |
| --- | --- | --- | --- | --- | --- | --- |

3. 我觉得VR很容易使用。 [单选题] *

| 非常不同意 | ○1 | ○2 | ○3 | ○4 | ○5 | 非常同意 |
| --- | --- | --- | --- | --- | --- | --- |

4. 我认为我需要技术人员的帮助才能使用VR。 [单选题] *

| 非常不同意 | ○1 | ○2 | ○3 | ○4 | ○5 | 非常同意 |
| --- | --- | --- | --- | --- | --- | --- |

5. 我觉得VR的各个功能整合得很好。 [单选题] *

| 非常不同意 | ○1 | ○2 | ○3 | ○4 | ○5 | 非常同意 |
| --- | --- | --- | --- | --- | --- | --- |

6. 我认为VR有太多地方相互矛盾。 [单选题] *

| 非常不同意 | ○1 | ○2 | ○3 | ○4 | ○5 | 非常同意 |
| --- | --- | --- | --- | --- | --- | --- |

7. 我想大多数人用很短时间就能学会使用VR。 [单选题] *

| 非常不同意 | ○1 | ○2 | ○3 | ○4 | ○5 | 非常同意 |
| --- | --- | --- | --- | --- | --- | --- |

8. 我觉得VR使用起来很麻烦。 [单选题] *

| 非常不同意 | ○1 | ○2 | ○3 | ○4 | ○5 | 非常同意 |
| --- | --- | --- | --- | --- | --- | --- |

9. 使用VR时，我感到很自信。 [单选题] *

| 非常不同意 | ○1 | ○2 | ○3 | ○4 | ○5 | 非常同意 |
| --- | --- | --- | --- | --- | --- | --- |

10. 大量的学习是熟练操作这个系统的必要条件。 [单选题] *

| 非常不同意 | ○1 | ○2 | ○3 | ○4 | ○5 | 非常同意 |
| --- | --- | --- | --- | --- | --- | --- |
