# Supplementary material for: Student perceptions toward virtual reality training in dental implant education
Source: PeerJ. 2023 May 5;11:e14857. doi: 10.7717/peerj.14857 (PMC10166074; doi:10.7717/peerj.14857)
Supplement: Supplemental Information 6 [file peerj-11-14857-s006.docx]

**VR技术和口腔医学教学结合的调查问卷**

十分感谢你参与本次VR体验。本问卷旨在了解学生对VR教学的体验感受，从而更好的将VR技术与口腔医学教学相结合，提高教学水平和质量。本问卷有22道题，大概需要5分钟时间完成，我们不会透露您的个人身份信息，再次感谢你的参与。

1. 你的年龄 [填空题] *

_________________________________

2. 你的年级 [单选题] *

| ○大一 |
| --- |
| ○大二 |
| ○大三 |
| ○大四 |

3. 你的性别 [单选题] *

| ○男 |
| --- |
| ○女 |

4. 你是否曾经使用过VR设备 [单选题] *

| ○是 |
| --- |
| ○否 |

**请针对你刚才体验的“种牙”VR学习过程，以下问题请你按照（0 =完全不同意；9 =完全同意）进行评价**

**Part1.体验感受**

5. VR操作简便易掌握 [单选题] *

| 完全不同意 | ○0 | ○1 | ○2 | ○3 | ○4 | ○5 | ○6 | ○7 | ○8 | ○9 | 完全同意 |
| --- | --- | --- | --- | --- | --- | --- | --- | --- | --- | --- | --- |

6. 即使没有助教的指引，我能独自使用VR学习实验教程。 [单选题] *

| 完全不同意 | ○0 | ○1 | ○2 | ○3 | ○4 | ○5 | ○6 | ○7 | ○8 | ○9 | 完全同意 |
| --- | --- | --- | --- | --- | --- | --- | --- | --- | --- | --- | --- |

7. 操作过程中我体验了虚拟环境下的触摸和震动等真实感觉。 [单选题] *

| 完全不同意 | ○0 | ○1 | ○2 | ○3 | ○4 | ○5 | ○6 | ○7 | ○8 | ○9 | 完全同意 |
| --- | --- | --- | --- | --- | --- | --- | --- | --- | --- | --- | --- |

8. VR手柄帮助我准确地体验了为患者种牙的操作。 [单选题] *

| 完全不同意 | ○0 | ○1 | ○2 | ○3 | ○4 | ○5 | ○6 | ○7 | ○8 | ○9 | 完全同意 |
| --- | --- | --- | --- | --- | --- | --- | --- | --- | --- | --- | --- |

9. 我刚才重复体验了这个操作过程。 [单选题] *

| 完全不同意 | ○0 | ○1 | ○2 | ○3 | ○4 | ○5 | ○6 | ○7 | ○8 | ○9 | 完全同意 |
| --- | --- | --- | --- | --- | --- | --- | --- | --- | --- | --- | --- |

10. 课上30分钟的VR时间对我来说足够掌握本节知识要点。 [单选题] *

| 完全不同意 | ○0 | ○1 | ○2 | ○3 | ○4 | ○5 | ○6 | ○7 | ○8 | ○9 | 完全同意 |
| --- | --- | --- | --- | --- | --- | --- | --- | --- | --- | --- | --- |

**Part2.系统评价**

11. 使用VR系统让我更有动力学习本节内容 [单选题] *

| 完全不同意 | ○0 | ○1 | ○2 | ○3 | ○4 | ○5 | ○6 | ○7 | ○8 | ○9 | 完全同意 |
| --- | --- | --- | --- | --- | --- | --- | --- | --- | --- | --- | --- |

12. VR系统让学习过程充满乐趣。 [单选题] *

| 完全不同意 | ○0 | ○1 | ○2 | ○3 | ○4 | ○5 | ○6 | ○7 | ○8 | ○9 | 完全同意 |
| --- | --- | --- | --- | --- | --- | --- | --- | --- | --- | --- | --- |

13. VR系统让学习更有效率 [单选题] *

| 完全不同意 | ○0 | ○1 | ○2 | ○3 | ○4 | ○5 | ○6 | ○7 | ○8 | ○9 | 完全同意 |
| --- | --- | --- | --- | --- | --- | --- | --- | --- | --- | --- | --- |

14. 相对于实验台上操作学习，我认为使用VR系统的学习成效更显著。 [单选题] *

| 完全不同意 | ○0 | ○1 | ○2 | ○3 | ○4 | ○5 | ○6 | ○7 | ○8 | ○9 | 完全同意 |
| --- | --- | --- | --- | --- | --- | --- | --- | --- | --- | --- | --- |

15. 我总体认为这种VR体验对我的学习有帮助。 [单选题] *

| 完全不同意 | ○0 | ○1 | ○2 | ○3 | ○4 | ○5 | ○6 | ○7 | ○8 | ○9 | 完全同意 |
| --- | --- | --- | --- | --- | --- | --- | --- | --- | --- | --- | --- |

**Part3.你的想法**

16. 就本节内容来说，我认为VR系统应该和传统实验相结合。 [单选题] *

| 完全不同意 | ○0 | ○1 | ○2 | ○3 | ○4 | ○5 | ○6 | ○7 | ○8 | ○9 | 完全同意 |
| --- | --- | --- | --- | --- | --- | --- | --- | --- | --- | --- | --- |

17. VR系统非常适合作为口腔实验课程的辅助工具。 [单选题] *

| 完全不同意 | ○0 | ○1 | ○2 | ○3 | ○4 | ○5 | ○6 | ○7 | ○8 | ○9 | 完全同意 |
| --- | --- | --- | --- | --- | --- | --- | --- | --- | --- | --- | --- |

18. 今天体验的VR系统看起来技术成熟，空间感真实。 [单选题] *

| 完全不同意 | ○0 | ○1 | ○2 | ○3 | ○4 | ○5 | ○6 | ○7 | ○8 | ○9 | 完全同意 |
| --- | --- | --- | --- | --- | --- | --- | --- | --- | --- | --- | --- |

19. 我认为VR系统会取代传统的口腔实验教学。 [单选题] *

| 完全不同意 | ○0 | ○1 | ○2 | ○3 | ○4 | ○5 | ○6 | ○7 | ○8 | ○9 | 完全同意 |
| --- | --- | --- | --- | --- | --- | --- | --- | --- | --- | --- | --- |

20. 我愿意花更多时间使用VR学习。 [单选题] *

| 完全不同意 | ○0 | ○1 | ○2 | ○3 | ○4 | ○5 | ○6 | ○7 | ○8 | ○9 | 完全同意 |
| --- | --- | --- | --- | --- | --- | --- | --- | --- | --- | --- | --- |

21. 体验了VR之后，您是否希望其他科学课程采用VR？ [单选题] *

| 完全不同意 | ○0 | ○1 | ○2 | ○3 | ○4 | ○5 | ○6 | ○7 | ○8 | ○9 | 完全同意 |
| --- | --- | --- | --- | --- | --- | --- | --- | --- | --- | --- | --- |

22. 总体而言，你如何评价这种VR体验？（自由回答题） [填空题] *

_________________________________
